# Functional skeletal muscle model derived from SOD1-mutant ALS patient iPSCs recapitulates hallmarks of disease progression

**DOI:** 10.1038/s41598-020-70510-3

**Published:** 2020-08-31

**Authors:** Agnes Badu-Mensah, Xiufang Guo, Christopher W. McAleer, John W. Rumsey, James J. Hickman

**Affiliations:** 1 NanoScience Technology Center, University of Central Florida, 12424 Research Parkway, Suite 400, Orlando, FL 32826 USA; 2 College of Medicine, Burnett School of Biomedical Sciences, University of Central Florida, Orlando, FL 32816 USA; 3 Hesperos Inc., 12501 Research Pkwy, Suite 100, Orlando, FL 32826 USA

**Keywords:** Mechanisms of disease, Muscle stem cells, Pluripotent stem cells, Nanobiotechnology, Stem-cell biotechnology, Tissue engineering, Diseases of the nervous system, Motor control

## Abstract

Recent findings suggest a pathologic role of skeletal muscle in amyotrophic lateral sclerosis (ALS) onset and progression. However, the exact mechanism by which this occurs remains elusive due to limited human-based studies. To this end, phenotypic ALS skeletal muscle models were developed from induced pluripotent stem cells (iPSCs) derived from healthy individuals (WT) and ALS patients harboring mutations in the superoxide dismutase 1 (SOD1) gene. Although proliferative, SOD1 myoblasts demonstrated delayed and reduced fusion efficiency compared to WT. Additionally, SOD1 myotubes exhibited significantly reduced length and cross-section. Also, SOD1 myotubes had loosely arranged myosin heavy chain and reduced acetylcholine receptor expression per immunocytochemical analysis. Functional analysis indicated considerably reduced contractile force and synchrony in SOD1 myotubes. Mitochondrial assessment indicated reduced inner mitochondrial membrane potential (ΔΨm) and metabolic plasticity in the SOD1-iPSC derived myotubes. This work presents the first well-characterized in vitro iPSC-derived muscle model that demonstrates SOD1 toxicity effects on human muscle regeneration, contractility and metabolic function in ALS. Current findings align with previous ALS patient biopsy studies and suggest an active contribution of skeletal muscle in NMJ dysfunction. Further, the results validate this model as a human-relevant platform for ALS research and drug discovery studies.

## Introduction

Amyotrophic lateral sclerosis (ALS) is an aggressive, multi-factorial disease characterized by progressive motoneuron degeneration, muscle weakness and wasting^1^. It typically manifests between ages 40 and 70 and results in death within 2–5 years of diagnosis. Ten percent of all reported ALS cases are familial (fALS) while the remaining are sporadic (sALS). Currently, genetic mutations in over twenty genes including superoxide dismutase 1 (SOD1), chromosome 9 open reading frame 72 (C9orf72), fused in sarcoma (FUS), and TAR DNA binding protein 43 (TDP43) are associated with ALS^2^. However, the pathogenesis of ALS remains largely unknown. It is understood that disruption of the neuromuscular junction (NMJ) is an early event in ALS pathology^3–6^, however, the mechanisms that lead to NMJ dysfunction are still unresolved. Specifically, there is disagreement in the literature regarding whether motoneuron (MN) dysfunction causes neuromuscular denervation, or skeletal muscle dysfunction drives synaptic degradation, thereby causes retrograde MN dysfunction and death^3,7,8^. The specific susceptibility of MNs in ALS led early researchers to adopt a “neurocentric” approach to investigating the disease^9,10^. However, mounting evidence suggests the involvement of the skeletal muscle in ALS onset via NMJ disruption^11–13^. Fischer and colleagues published evidence of disrupted NMJs without signs of MN death in a spatiotemporal study performed in ALS murine models^5,6,12^. In the same report, they presented pathology results from an ALS patient that demonstrated skeletal muscle atrophy without MN cell death^5^. In another, the muscle-specific overexpression of mutant human SOD1 protein was stated to be sufficient to drive disease symptoms in a transgenic mouse model^14,15^. Since then, many investigators have reported observations of skeletal muscle metabolic dysregulation and atrophy before MN degeneration in ALS animal models^16,17^. In a study by Deloach et al., they reported that ALS skeletal muscle secreted axonal chemorepellent molecules such as Nogo-A, that stunt axonal growth^18^. Also, there are reports of altered morphology and regenerative capabilities of satellite cells derived from patient skeletal muscle biopsies^19,20^. Despite the above-mentioned findings, the role of skeletal muscle dysfunction in ALS remains elusive due to the scarcity of human-based studies that substantiate the phenomenon, the presence of other cell types that complicate the investigation of muscle pathology, and the lack of models that can reproduce the evolvement of muscle pathology from the beginning of muscle development.

This study aimed to investigate the regenerative and functional deficits of the ALS skeletal muscle by developing a functional in vitro phenotypic skeletal muscle model from ALS patient-derived iPSCs (ALS-iPSCs) harboring mutations in the SOD1 gene. This patient iPSC-derived muscle model is human-based, free of other cell types such as motoneurons, and allows the investigation of muscle pathology from myogenesis to functional muscle formation. With the iPSC-derived model, we demonstrated that ALS-iPSC myoblasts have deficits in fusion despite their expression of appropriate myogenic markers. Additionally, significant morphological and structural alterations were identified in iPSC-derived ALS myotubes that correlated with decreased contractile and metabolic function. Furthermore, subcellular investigation revealed ALS skeletal muscle had altered mitochondrial function, which may negatively impact metabolic pathways and energy generation. Compared to previous iPSC-derived muscle studies, this investigation provides a detailed and comprehensive view of the morphological and structural deformity of ALS muscle and demonstrates for the first time their functional defects in contractibility, as well as their metabolic dysregulation. The abnormalities revealed in these patient iPSC-derived muscle models indicates that endogenous expression of the mutant SOD1 gene in muscle, independent of the influence of motoneurons, has a toxic effect on skeletal muscle regeneration and function, which supports the active role of muscle in NMJ degradation and ALS onset and/or progression.

## Results

### Characterization of WT and SOD1-mutant iPSC myoblast lineage determination

**Figure 1:**
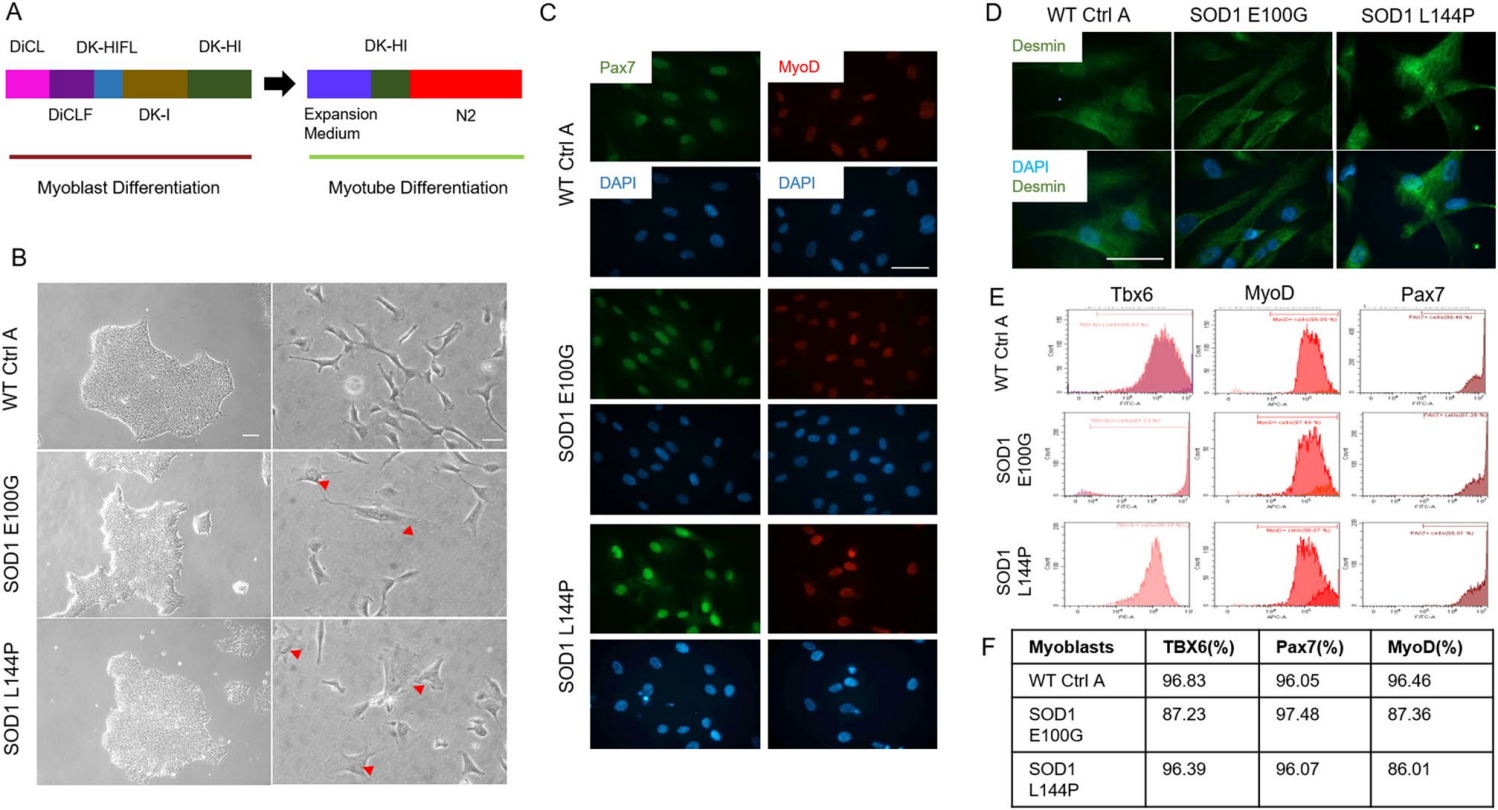
Myoblast derivation and myogenic characterization. (**A**) Illustration of the differentiation protocol used in both myoblast and myotube phases of differentiation. (**B**) (left panel) Phase contrast images of iPSC cultures of the various cell lines in maintenance medium (scale bar = 100 μm). (**B**) (right panel) Phase contrast microscopy images of resultant myoblast cultures after replating in proliferation medium at the end of the myoblast differentiation phase (scale bar = 50 μm). Red arrowheads directed at flat myoblasts in SOD1 cultures. (**C**) Immunocytochemistry (ICC) staining of derived myoblasts for Pax7 and MyoD expression across all cell lines (scale bar = 50 μm). (**D**) ICC staining for Desmin expression in WT and SOD1 cultures. (**E**) Flow cytometry (FC) result plots showing TBX6 expression during the first week of differentiation (left panel) and PAX7 and MyoD expression by the third week of myoblast differentiation (middle and right panels respectively). Data analyzed with CytExpert software, version 2.1 (Beckman Coulter). (**F**) Tabulated percentage values of FC results.

Three iPSC lines, wild type control A (WT Ctrl A), superoxide dismutase (SOD1) mutation with glutamate 100 to glycine switch (E100G) and SOD1 with lysine 144 to proline switch (L144P) were differentiated into myogenic progenitors (myoblasts) by the simultaneous activation of WNT signaling and inhibition of bone morphogenetic protein (BMP) pathways using a serum-free, small molecule-directed protocol adopted from Chal et al. (Fig. 1A)^21^. Experiments that led to the modifications to Chal’s protocol are summarized in Supplementary Table S1. Correct iPSC to myogenic lineage progression was confirmed and quantified by evaluating the timely expression of known differentiation markers including T-box transcription factor 6 (TBX6), myoblast determination protein 1 (MyoD) (proliferative satellite cell marker) and paired box protein 7 (Pax7) (satellite cell marker) via flow cytometry (Fig. 1D). WT, SOD1 E100G and L144P cultures were found to have comparable percentages of cells expressing each myogenic marker (Fig. 1E,F). At the end of the myogenic progenitor differentiation, myoblasts from all three lines were seeded at low density for morphological assessment via phase-contrast and fluorescent microscopy. While WT myoblasts were generally spindle-shaped, the SOD1 myoblasts were a mix of spindle-shaped and flat-like, irregularly shaped cells (Fig. 1B). Regardless of morphology, immunocytochemistry results indicated that WT and SOD1 myoblast cultures were MyoD and Pax7 positive confirming the flow cytometry data (Fig. 1C). Together, these data indicated that myoblasts derived from healthy and ALS-mutant PD-iPSCs were proliferative and expressed markers essential for correct myogenic progression differentiation.

### Characterization of WT and SOD1-mutant myogenic progenitor fusion

**Figure 2:**
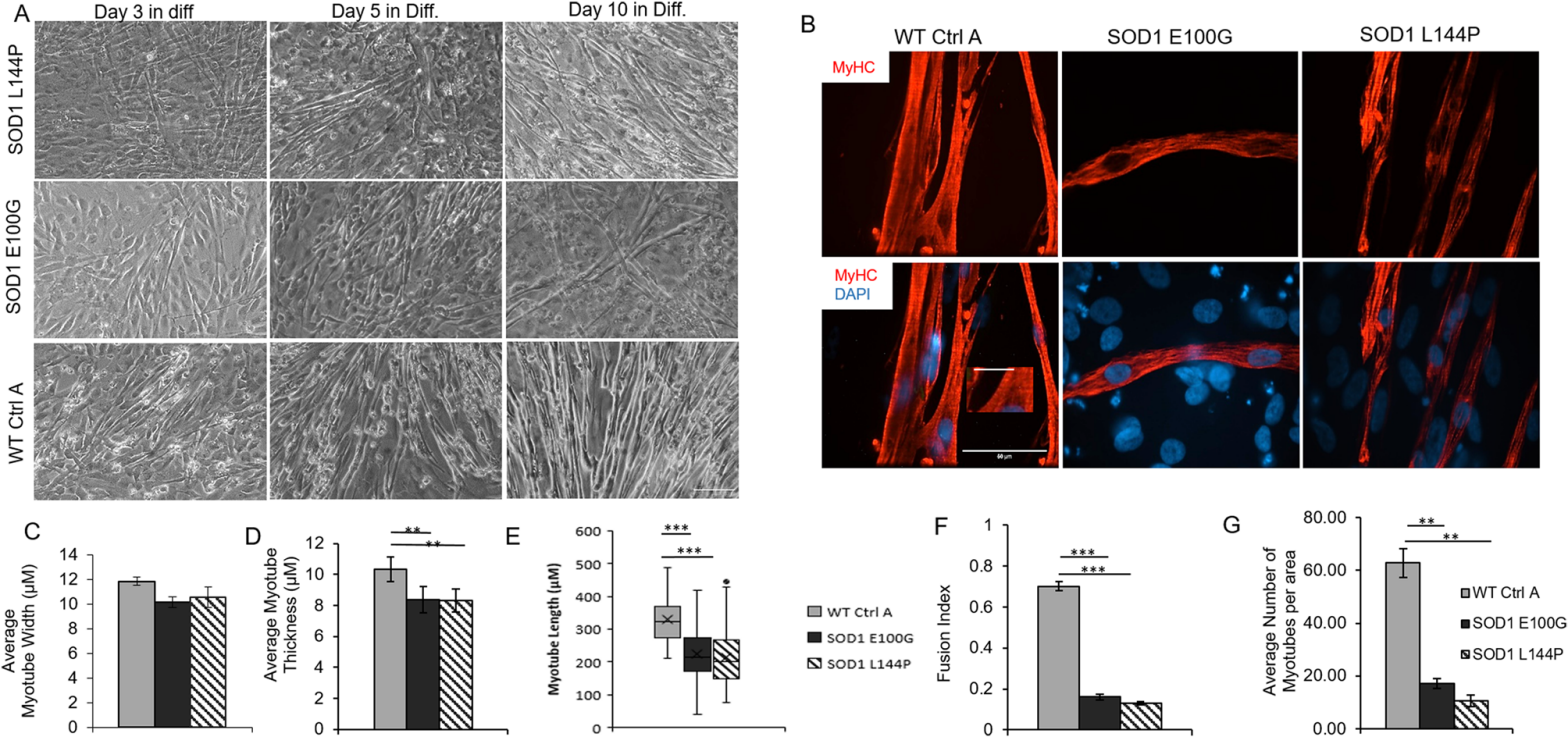
Phenotypic myotube characterization. (**A**) Phase contrast images showing myotube derivation from myoblasts from the three cell lines. Scale bar = 100 μm. (**B**) ICC images of myotube cultures stained for MyHC and DAPI. Note the unfused background cells in the DAPI^+^/MyHC SOD1 cultures. Imaged with Volocity software, version 6.3.0 (PerkinElmer). Scale bar = 50 μm. (**C**) Bar graph showing slightly decreased myotube cross sectional width compared in SOD1 cultures relative to WT (Error bars CI = 90%). (**D**) Average myotube thickness **p value = 0.018. (**E**) Box and Whisker plots describing the lengths of WT and SOD1 myotubes. ***p value < 0.001. One-way ANOVA, followed by Dunnett’s test. (**F**) Bar graph showing fewer fusion events in the SOD1-mutant cultures compared to WT, evidenced by fusion index. (**G**) Bar graphs indicating lower average myotube number per area in SOD1 cultures compared to WT. Error bars: SEM *p < 0.1; **p < 0.01; ***p < 0.001 by Student T-test, 2 tails, unequal variance.

The ability of satellite cells to initiate proliferation and progress through the myogenic differentiation program is essential to skeletal muscle repair and mass maintenance after injury^22^. Thus, SOD1 myoblasts were tested for their ability to differentiate into myotubes. While WT cultures had nascent myotube formation on day three after switching to differentiation medium, none were observed in SOD1 cultures until the fifth day in differentiation medium (Fig. 2A). Cultures were maintained for 14 days to accommodate the slower differentiation of the SOD1-mutant cultures. On the 14th day of differentiation, the fusion indices of all cultures were quantified (Fig. 2B). Compared to WT, SOD1-mutant myoblasts had a significantly lower fusion index (*p* < 0.001) (Fig. 2F). Additionally, the number of myotubes per area in each culture was quantified as another measure of fusion efficiency. While WT cultures were dense with myotubes, SOD1-mutant cultures had 3× less myotubes (Fig. 2G). To ensure that the decreased fusion in the mutant cultures was not due to the presence of non-myogenic cells, the culture was stained with the myogenic-specific marker, desmin. Both myotubes and unfused myoblasts in the cultures stained positive for desmin (Fig. S1). The results confirmed that the observed reduced fusion in ALS cultures was not due to the presence of contaminating cells, but the inability of the myoblasts to progress through the differentiation program. Altogether, ALS-mutant cultures exhibited altered/decreased fusogenicity compared to WT cultures.

### Morphometric assessment of WT and SOD1-mutant myotubes

**Figure 3:**
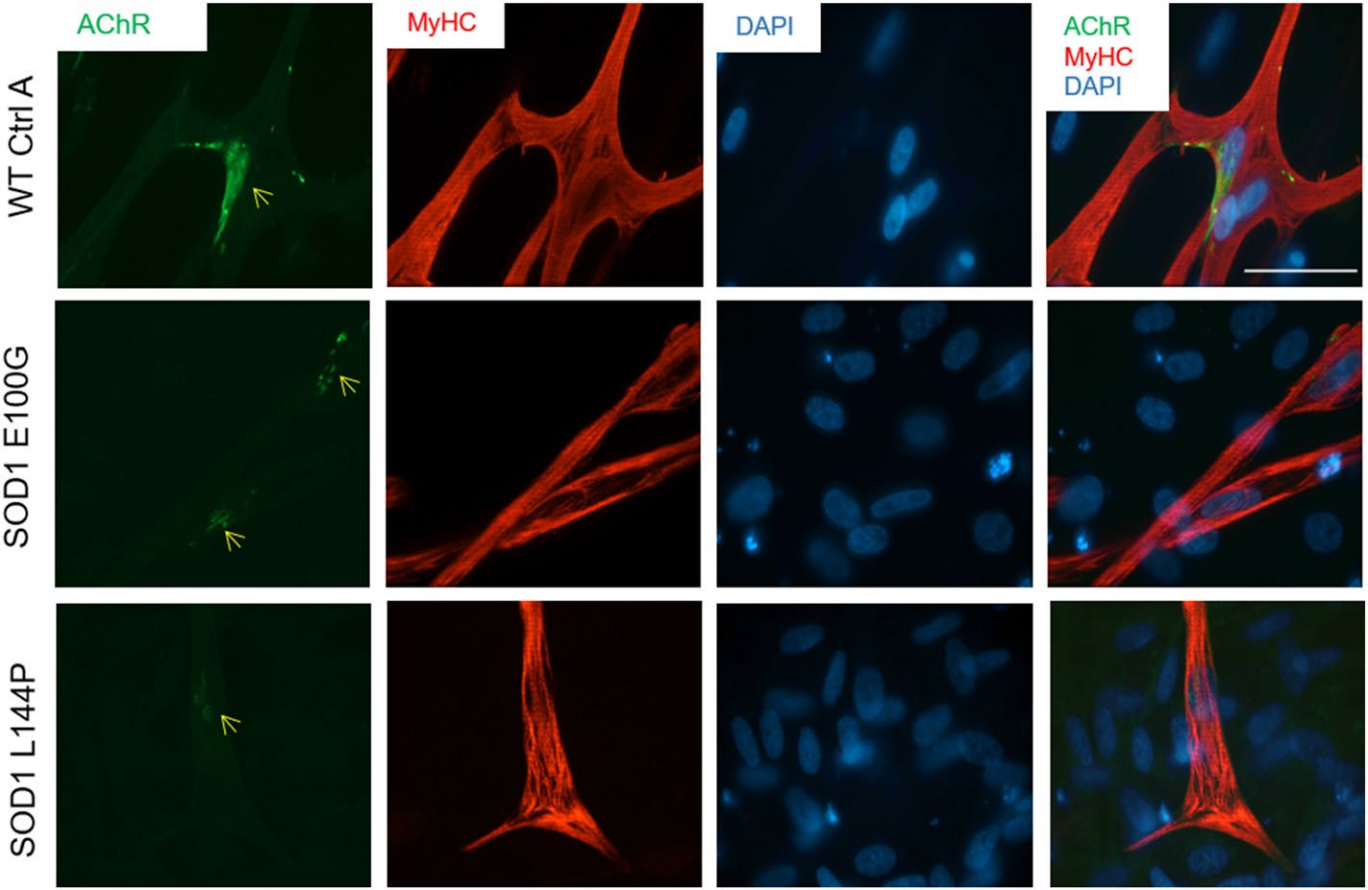
Phenotypic myotube characterization. Myotubes stained for MyHC, nAChR and DAPI, depicting diffuse to no AchR clusters in SOD1-mutant cultures. Yellow arrows point to nAChR clusters on SOD1 myotubes. Nuclei staining (DAPI) showing decreased fusion in myotube cultures, as evidenced by MyHC^−^/DAPI^+^ unfused myoblasts in the background. Imaged with Volocity software, version 6.3.0 (PerkinElmer). Scale bar = 50 μm.

The phenotypic characteristics and structural organization of SOD1 myotubes were evaluated to delineate morphometric alterations when compared to WT. Measurements of the average width, thickness and length of myotubes from each cell line were assessed. While SOD1 myotubes were slightly narrower than WT myotubes, they were significantly thinner and shorter (*p* = 0.018, < 0.001 respectively) (Fig. 2C–E). The complex spatial organization of myogenic-proteins is vital to their ability to accurately execute function^23^. Thus, the structural organization of Myosin Heavy Chain (MyHC) within myotubes from each culture, as well as nicotinic acetylcholine receptor (nAChR) clustering, were analyzed via immunocytochemistry. MyHC appeared densely arranged and compact in the WT myotubes. Additionally, WT myotubes expressed nAChR clusters throughout the culture (Fig. 3). Conversely, SOD1 myotubes had disorderly and loosely arranged MyHC that made striations within individual fibers appear discontinuous. Also, there were few to no nAChR clusters on SOD1 myotubes, and nAChR staining appeared diffuse and faint (Fig. 3). Collectively, these results demonstrate that SOD1-mutant myotubes have altered morphological and structural deficits that may affect function.

### Functional evaluation of WT and SOD1-mutant myotubes

**Figure 4:**
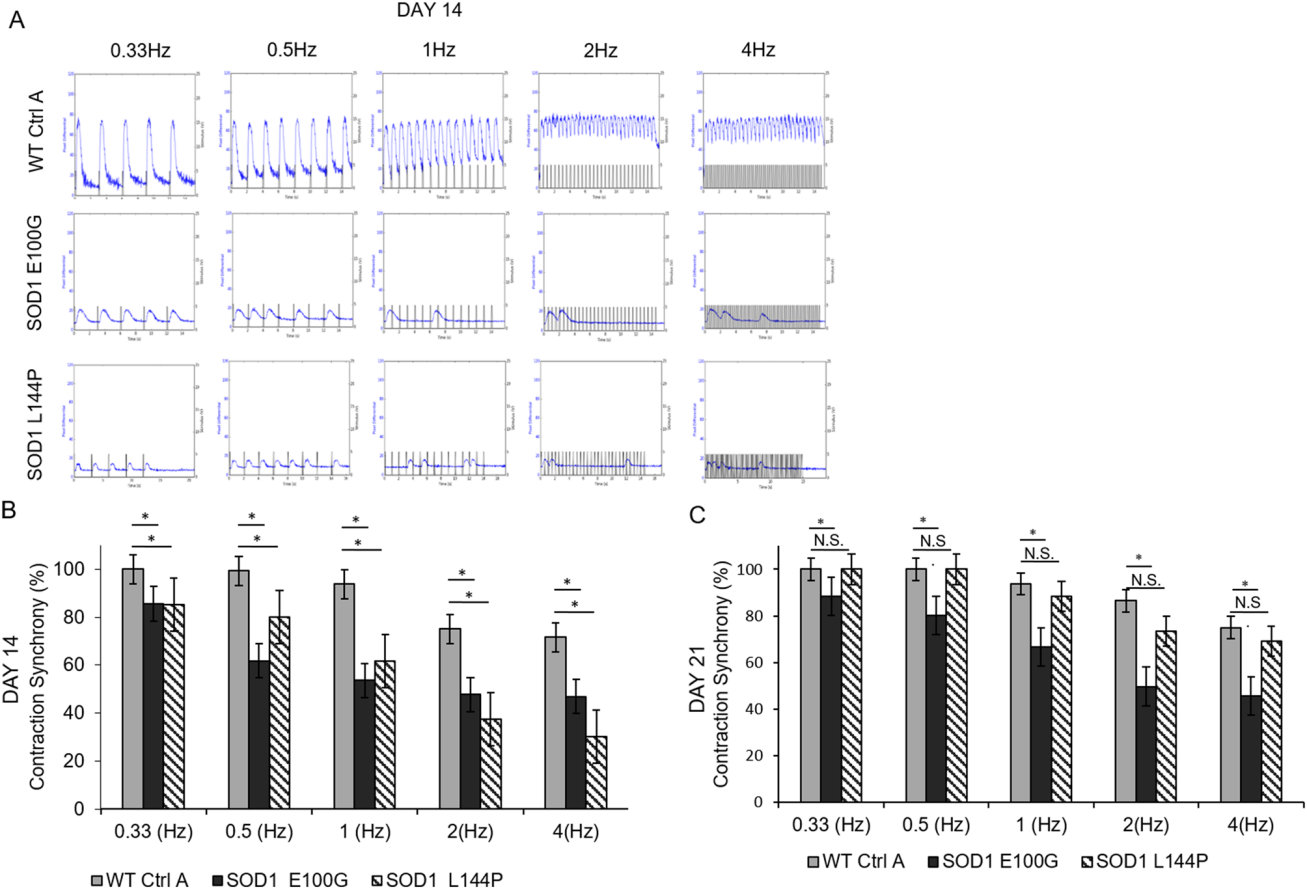
Functional characterization of myotubes. (**A**) Contraction traces of individual myotubes from iPSC-derived SOD1 E100G, SOD1 L144P and WT control cultures at 0.3, 0.5, 1, 2 and 4 Hz, on day 14 of myotube differentiation. Electrical pulses were elicited at 1 V for 15 s. (**B**, **C**) Bar graphs showing the percentage of contraction synchrony at days 14 and 21. *p* ≤ 0.05 ANOVA on ranks followed by Holm-Sidak method. *NS* Not significant.

**Figure 5:**
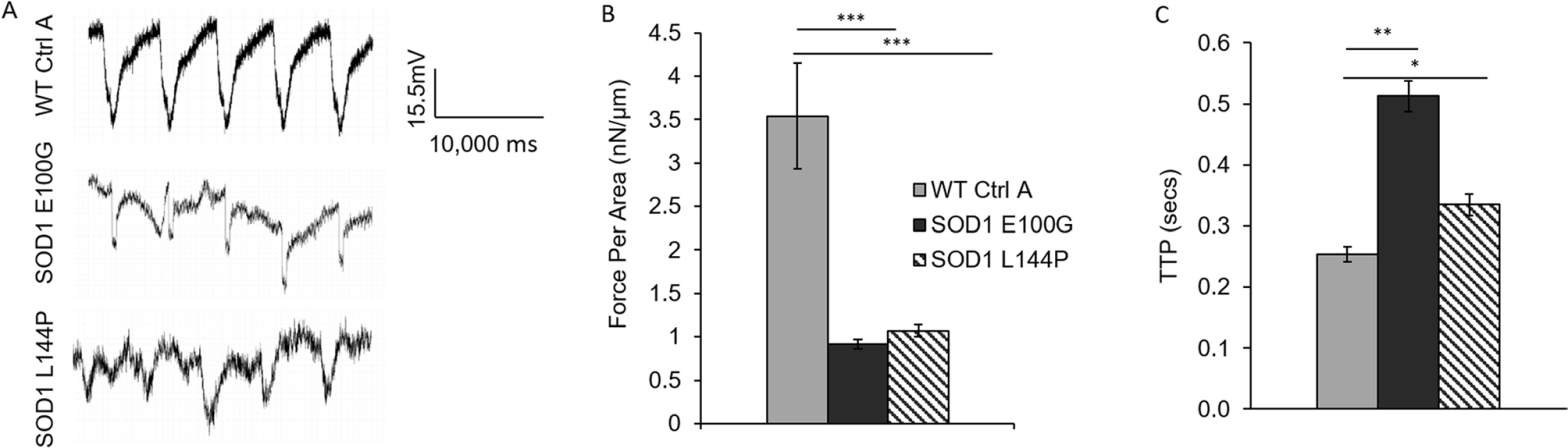
Functional characterization of myotubes. (**A**) Representative cantilever traces of WT and SOD1 myotubes. Traces extracted with Clampfit Version 10.7 (Axon Instruments). (**B**) Bar graph showing the contractile force per area of the respective WT and SOD1 muscle lines. (**C**) Results depicting the time-to-peak force of WT and SOD1 muscle. *p < 0.1, **p < 0.01, ***p < 0.001, One-way ANOVA, followed by Dunnett's test.

Muscle weakness is an established ALS hallmark and has been described in both patients and transgenic animal models^24,25^. From the neurocentric viewpoint, muscle wasting and weakness are a result of motoneuron denervation. However, this opinion has been challenged by recent findings that postulate there are pathological alterations in diseased skeletal muscle function devoid of axonal retraction^5,8,26^. To test the latter hypothesis, individual myotubes from all lines were subjected to stimulated contraction at frequencies of 0.3, 0.5, 1.2 and 4 Hz on day 14 of differentiation (Fig. 4A) and the functional activity of WT myotubes was compared to that of SOD1 myotubes. WT myotubes contracted consistently with increasing frequency, however both SOD1 E100G and L144P myotube twitches were asynchronous and had comparatively weaker amplitudes (Fig. 4A,B). As expected, contractile force output for SOD1 myotubes was also comparatively low (Fig. 5A,B). Additionally, to examine the dynamics of myofiber contraction, time-to-peak (TTP) was analyzed by quantifying the duration from the initiation to the peak of the contraction under electrical stimulation. As shown in Fig. 5C, there was significantly higher contraction time-to-peak for both SOD1 E100G and L144P compared to WT, indicating a slowdown of contraction dynamics. Because the SOD1 myotubes has been shown to mature at a slower pace, myotubes were tested at day 21 of differentiation. This was done to determine if a longer maturation period could improve contractile synchrony and amplitude in the SOD1 cultures. While the contractile synchrony in most SOD1 L144P myotubes improved by day 21, the amplitude remained the same (Fig. 4A–C) (Supplemental Fig. S2). Conversely, SOD1 E100G cultures indicated no improvement in either contraction synchrony or amplitude with increased time in culture (Fig. 4A–C) (Supplemental Fig. S2). These results suggest that SOD1 myotubes have intrinsic defects that retard maturation and contractile function independent of denervation.

### Mitochondrial function analysis of WT and SOD1-mutant myotubes

**Figure 6:**
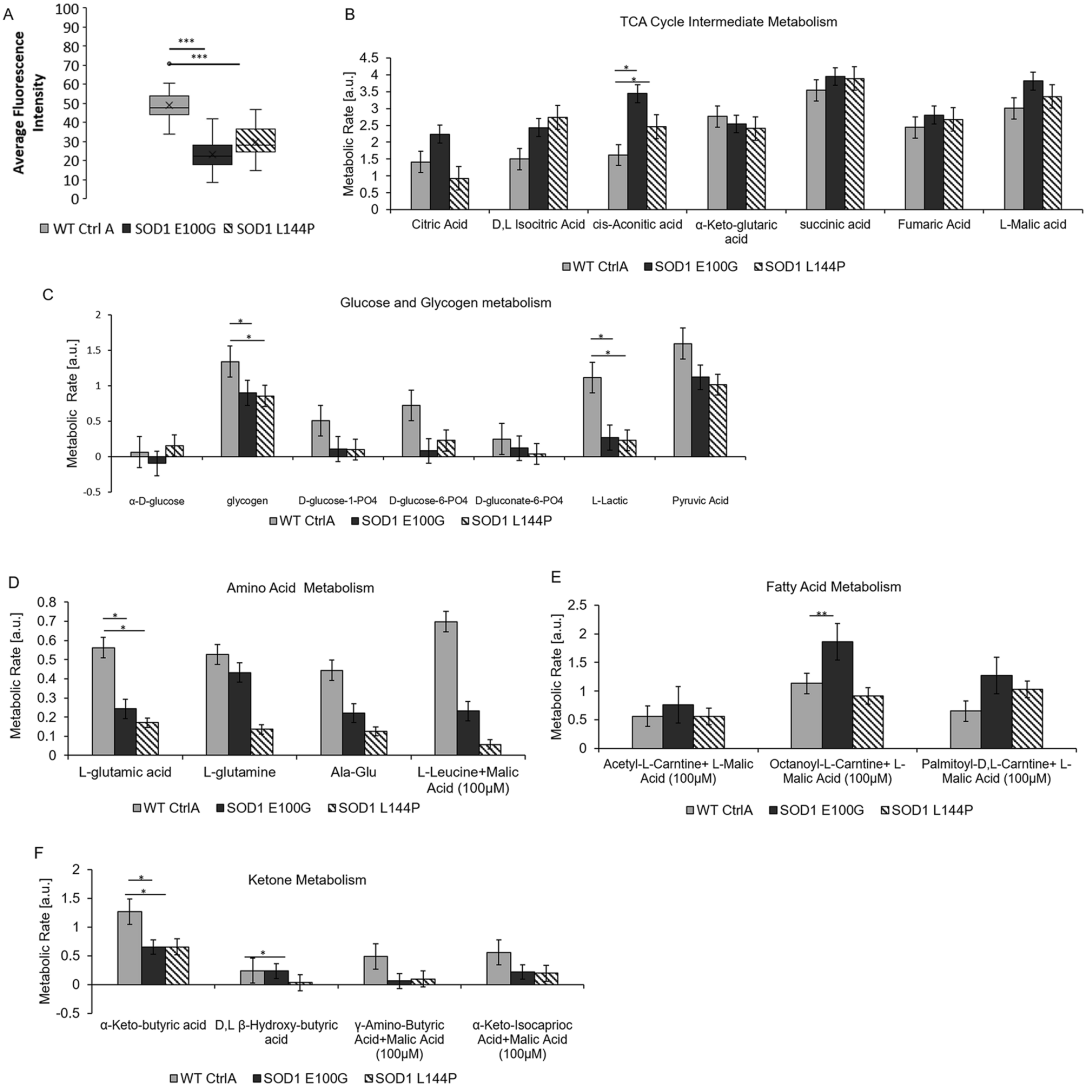
Mitochondria fsunctional assessment. (**A**) TMRE assay measuring fluorescence intensity as a measure of mitochondrial membrane potential at day 12 of differentiation. SOD1-mutant muscle exhibit decreased mitochondrial membrane potential compared to WT Ctrl A. Error bars: SEM *p < 0.1; **p < 0.01; ***p < 0.001 by Student T-test, 2 tails, unequal variance. (**B**, **C**) Mitochondrial metabolic rate assessment of SOD1 muscle for TCA cycle intermediates and glucose-derived substrates. (**D**–**F**) Mitochondrial metabolic rate assessment of SOD1 muscle for amino acid-, fatty acid- and ketone-derived substrates respectively.

Studies have reported changes in mitochondrial morphology, dynamics and function in ALS pathology^4,24,27,28^. Additionally, the processes of muscle fusion and contraction are energy-intensive activities^29,30^. Thus, the mitochondrial function of SOD1 cultures was investigated as a possible subcellular mechanism for the phenotypic observations in muscle regenerative and contractile function. The mitochondrial membrane potential (ΔΨm) of diseased and WT myotubes was assessed because the ΔΨm has been shown to be vital to a myriad of cellular processes including energy production, calcium handling and DNA repair^31,32^. Results indicated significantly lower average ΔΨm values for both SOD1 myotube lines compared to WT (Fig. 6A). Mitochondrial function of the SOD1 myotubes was evaluated by measuring the rate of metabolism of various substrates. Overall, tricarboxylic acid (TCA) cycle intermediates and fatty acids were metabolized at either a slightly faster or the same rate by SOD1 mitochondria compared to WT (Fig. 6B,C). However, SOD1 cultures metabolized amino acids, ketones and glycogenic substrates at a slower rate compared to WT (Fig. 6D–F). Among the assayed substrates, lactate metabolism was particularly low in diseased cultures compared to WT (Fig. 6B). Together, these results suggest that mitochondrial dysfunction is a characteristic of SOD1 mutant skeletal muscle.

## Discussion

The goal of this study was to create a phenotypic model of ALS muscle to investigate the inherent defects of the ALS skeletal muscle in regeneration and function to better evaluate their contribution to NMJ disruption in ALS. To achieve this, functional in vitro biomimetic ALS skeletal muscle models were developed from fALS patient-derived iPSCs. Two patient iPSCs lines harboring mutations in the SOD1 gene resulting in amino acid substitutions at L144P and E100G, were differentiated into skeletal muscle myoblasts and subsequently myotubes. Systematic analysis of the ALS myoblasts revealed deficits in myogenesis, myotube morphology, structure and function, as well as mitochondria properties and metabolism. Myotubes had notably decreased acetylcholine receptor (AChR) clusters and loosely arranged myosin heavy chain proteins compared to WT myotubes. BioMEMs multiplexed cantilever analysis indicated ALS-iPSC myotubes have significantly weaker force output and reduced contraction synchrony compared to WT controls. These defects identified in iPSC-derived ALS muscle, in the absence of other cell types, indicate muscle is an active contributor to the initiation and/or progression of NMJ dysfunction in ALS.

ALS-iPSC myoblasts were found to be proliferative and expressed the appropriate stage-specific myogenic markers at percentages comparable to WT. However, the flat and stretched morphology observed in the ALS myoblast cultures reiterates the senescent-like morphology reported in a patient myoblast study by Pradat et al.^19^. We also demonstrated that ALS-iPSC myoblasts exhibited delayed and decreased fusibility when subjected to differentiation. It has been established that correct myoblast activation and fusion are pertinent to skeletal muscle mass maintenance and repair^33^. Thus, any impairment in the myogenic developmental program may have adverse effects on downstream regeneration. Most SOD1 myoblasts were unable to fuse upon induction, suggesting there may be defects in skeletal muscle regeneration in ALS pathology. The diminished fusibility of SOD1 myoblasts observed in the current model could affect muscle mass maintenance and repair in patients, which may partly explain observed muscle atrophy. This has been reported in previous patient skeletal muscle biopsy studies^19,20^.

Morphometry results showed that the mutant myotubes had narrower widths and shorter lengths compared to WT controls. At a higher magnification, MyHC proteins appeared loosely arranged in SOD1 myotubes than in WT. Contrastingly, Pradat et al., reported that ALS myotubes were long and thin, while the SOD1 myotubes in our cultures were shorter and narrower than WT^19,20^. Notwithstanding, immunocytochemistry results from both labs demonstrated lower expression level of MyHC in ALS myotubes. This could explain the loose arrangement of MyHC and reduced thickness of myotubes observed in our culture system^19,20^.

Another notable observation was the reduced expression of AChR on SOD1 myotubes compared to WT myotubes. AChR clusters expressed on ALS-iPSC myotubes were smaller and appeared diffuse compared to the WT (Fig. 3). Robust expression and dense clustering of AChRs are necessary to receive action potential signals from the presynaptic motoneuron. Reduced expression and clustering decrease the possibility for accurate transmission of motoneuron signaling events. Abnormal AChR function has been described in both patient samples and mice models^34,35^. Recently, Picchiarelli et al. reported decreased AChR clustering and endplate maturation in FUS-mutant skeletal muscle generated from patient iPSCs as possible cause of NMJ dysfunction^36^.

In addition, integration of ALS muscle with BioMEMs cantilevers revealed cell-autonomous deficits in contractile force output and time-to-peak force. Although slowed contraction has been reported in the SOD1 G93A transgenic murine model, this is the first human-based work to directly demonstrate defective contractile function of ALS muscle in a human-based system^37^, although a previous researcher did extrapolate functional defects by measuring the passive electrical properties of ALS skeletal muscle generated from patient iPSCs^38^. While skeletal muscle weakness is an established hallmark of ALS, the exact cause of this dysfunction is unclear. The generally accepted hypothesis is that muscle weakness in ALS is a result of MN denervation and axonal retraction^39^. However, contrary to this view, muscle-centric studies suggest an intrinsic cause of muscle weakness^14,15,40^. In the current model, ALS-iPSC myotubes exhibited over 70% lower contractile force compared to WT. Evaluation of the time-to-peak force (TTP) showed a significantly delayed TTP in SOD1 myotubes (Fig. 5C). This observation is suggestive of irregularities in the excitation–contraction-coupling (ECC) machinery and/or calcium regulation. It is noteworthy that while pathological ALS MNs could be an inevitable component of muscle weakness in vivo, this study provides solid evidence confirming significant innate muscle deficits independent of motoneurons. By reproducing human myogenesis from the stem cell stage, this systematic study revealed that ALS myotubes have deficits in myotube regeneration, the capability of receiving innervation, contractibility upon excitation, and in the force of contraction. It is conceivable that the combination of these muscle-derived deficits and those from neuron-derived components result in the muscle weakness symptoms seen in ALS patients.

Furthermore, we identified mitochondrial dysregulation in these ALS muscle. First, TMRE staining indicated a significantly decreased ΔΨm in SOD1 cultures compared to WT myotubes. ΔΨm is known to play a pivotal role in oxidative phosphorylation, therefore, a reduced ΔΨm would imply the inability of ALS skeletal muscle to provide energy support for its function^31^. More importantly, decreased ΔΨm interferes with Ca^2+^ handling, which is an important regulator of aerobic respiration^31,32^. Decreased ΔΨm has also been observed in the muscle of SOD1 mice models in the absence of axonal retraction, suggesting skeletal muscle dysfunction could precede NMJ disruption^27^. Additionally, altered mitochondrial morphology, dynamics and function have been reported in ALS patient muscle studies and murine models^27,28,41^.

We also demonstrated that ALS-iPSC myotubes have reduced metabolic plasticity. With the provision of citric acid cycle intermediates or fatty acids, the rate of metabolism in SOD1 cultures was either the same or had a slightly faster rate compared to WT. However, metabolism of glycogen, lactate, amino acids, and ketones were relatively slower in SOD1 cultures. The current findings are in line with previous studies that reported a reduced preference for glycolytic metabolites and enhanced lipid metabolism in ALS skeletal muscle^42–44^. In addition to altered metabolism, researchers have also reported a shift toward an oxidative fiber type composition without apparent alterations in MyHC composition and expression^6,42,44^. The heterogeneity in metabolite preference among skeletal muscle fiber types is a known phenomenon. For instance, fast twitch fibers typically prefer the use of glucose for ATP production via anaerobic respiration. In contrast, slow twitch fibers tend to utilize fatty acids for ATP synthesis^45^. The ability of diseased skeletal muscle to efficiently metabolize fatty acids may explain the resilience of slow twitch fibers during ALS pathology^6^. Further investigation will be needed to elucidate the relationship between fiber type and metabolism alterations during ALS progression, and this topic can be pursued in future studies.

The inability of ALS-iPSC muscle to metabolize lactate stood out among all glucogenic substrates. Once thought to be a by-product, lactate has more recently been found to be major energy source for skeletal muscle. Reduced lactate turnover has been reported in fALS patients and lactate metabolism was observed to significantly decrease with disease progression^46^. Consistent reports concerning mitochondria defects in muscle phenotypes from different ALS models suggest a mechanistic link between these two pathologies. While the mechanism of the observed changes in ALS mitochondrial function is unclear, the results imply that provisions for selective nutrient substrate utilization could partially circumvent the muscle metabolism obstacle and possibly alleviate this pathology. This idea is supported by the effectiveness of a holistic therapy, the Deana Protocol, which contains the citric acid cycle intermediates as a supplement^47^.

While there is documented evidence of skeletal muscle dysfunction in ALS patients, exactly how the underlying ALS-associated genetic mutations induce dysfunction is poorly understood, which makes outlining the pathogenic role of the muscle challenging. For instance the SOD1 mutants E100G and L144P, both with substitutions distant from the metal-binding region of the protein, generated similar phenotypes but with significant variation in the severity level of functional and metabolic defects^43,48^. Both mutations are missense substitutions located in exons 4 (E100G) or 5 (L144P). However, it is hard to speculate further concerning the molecular pathology due to the limited information available for these molecular changes due to these two mutations. Genetic and phenotypic heterogeneity is well-known in SOD1 mutations. Over 180 different SOD1 mutations have been identified throughout the five exons of the SOD1 gene. Of these, more than 160 have been reported to be disease-associated. However, the precise shared and/or differing molecular and cellular pathological mechanism of each SOD1 mutation has yet to be elucidated^49,50^. Additionally, the genetic background of the subjects adds another significant variable^19,20^. The ALS-iPSC skeletal muscle models examined in this study demonstrates deficits in the ALS skeletal muscle by directly measuring its regenerative, contractile and metabolic functions. This phenotypic model, by performing systematic characterization of the functional changes in skeletal myofibers containing patient-specific mutations, constitutes a valuable new platform for addressing this issue by providing the gene-phenotype linkage.

Overall, we have developed a patient iPSC-derived skeletal system that recapitulates typical ALS pathology in both phenotypes and also for subcellular mechanisms. Because this is a phenotypic model allowing for the quantification of muscle function, it could be utilized in high content drug toxicological and efficacy screening. In addition, when coupled with Human-on-a-chip and BioMEMs technologies, it may be used in engineering physiologically relevant multi-organ models for studying complex interactions among tissues during ALS onset and progression. Compared to patient biopsy samples, sourcing from iPSCs eliminates the limitation for the cell source availability and enables the possibility of generating multiple cell types from the same iPSC, patient-specific models. Collectively, this system provides a valuable platform for ALS translational studies and therapeutic development.

## Conclusion

In conclusion, this work demonstrates that ALS patient-derived skeletal muscle has inherent defective regenerative capabilities and function independent of motoneuron influence or denervation. This patient iPSC- derived SOD1 skeletal muscle model recapitulates most ALS-diseased muscle characteristics reported in human clinical and transgenic animal studies. Thus, this iPSC-derived muscle system is a good resource for etiological investigations and relevant high content drug testing.

## Materials and methods

### Cantilever fabrication and surface chemistry engineering

Cantilever microelectromechanical systems (MEMS) were made from 6-inch silicon-on-insulator wafers, polished with a 5 μm silicon layer and a 500 μm silicon dioxide layer on the front and back respectively, as previously described^51^. The cantilever patterning was done using S1818 photoresist, followed by deep reactive ion etching. Glass coverslips were soaked in 12 M hydrochloric acid and placed on a shaker overnight. The next day, the coverslips were thoroughly rinsed with sterile water, and transferred to 100% ethanol. After 24 h, the coverslips were allowed to dry overnight and stored in a desiccator.

### IPSC-derived myoblast differentiation

Human induced pluripotent stem cell (iPSCs) lines ND41865, ND35662, ND39032 were obtained from the Coriell Institute for Medical Research. iPSCs were passaged a maximum of 12 times per recommended NIH passaging protocols. Cells from passages 6 to 12 were used in myoblast differentiation experiments. Myoblast differentiation was performed strictly following a protocol published by Chal et al.^21^. Briefly, iPSC cultures were maintained in mTESR1 (Cat# 85850, StemCell Technologies) or TESR-E8 (Cat# 05990, StemCell Technologies) on matrigel-coated (Cat# 354230 Corning Life Sciences) surfaces to a 15–20% confluence before commencing myoblast differentiation. Cultures were maintained until 35–40 days of differentiation and harvested. At the end of myoblast differentiation, the cultures were re-plated at 1:6 in MyoCult serum free supplemented 1000 mg/L DMEM (Cat #05982; StemCell, Technologies) for myoblast expansion. Medium was refreshed every other day until 50–60% confluence. Myogenic progenitors were harvested and cryopreserved for downstream applications. All cultures were maintained in humidified air supplemented with 5% CO_2_, at 37 °C.

### IPSC-derived myotube maturation

Myoblasts were plated on acid-washed coverslips in Myocult medium (1000 mg/L DMEM supplemented with MyoCult Serum Free nutrient cocktail) until confluent. Next, cultures were switched to DK-HI medium (DMEM/F12 21041-025 ThermoFisher Scientific, 15%vol/vol Knockout Serum Replacement ThermoFisher Scientific 10828-028, 10 ng/ml hepatocyte growth factor (HGF) Peprotech 315-23, 2 ng/ml insulin-like growth factor (IGF), Sigma I1271, 0.1 mM β-mercaptoethanol, ThermoFisher Scientific 31350010, 1% MEM Non-Essential Amino Acids Solution ThermoFisher Scientific 11140050 and 100 nM dexamethasone, Sigma D4902). After two days, cultures were switched to the terminal differentiation medium, N2 (DMEM/F12 21041-025 ThermoFisher Scientific, 1% Insulin Transferrin Selenium (ITS) 41400045 ThermoFisher Scientific, 1% N2 supplement 17502048, ThermoFisher Scientific, 1% l-glutamine 20 mM A2916801 ThermoFisher Scientific, 100 nM dexamethasone, Sigma D4902).

### Myoblast myogenic characterization

Cultures, during the course of myoblast differentiation, were subjected to flow cytometry (FC) to confirm temporal activation of the myogenesis gene program. Differentiation markers assessed were the pre-somite marker, TBX-6 (Abcam ab170482), after 4–8 days of differentiation and the myogenic progenitor markers, Pax7 (Invitrogen PA5-117P) and MyoD (Santa Cruzsc77460; LS Bio LS C263888), after 18–21 days in differentiation. Differentiating cultures were lifted using TrypLE Express or Trypsin–EDTA 0.05% (ThermoFischer Scientific12605010, 25300062) and centrifuged at 280 g for 5 min. The resulting pellet was re-suspended in FC buffer (10% BSA). An equal volume of 4% paraformaldehyde, (PFA) in 1X phosphate buffered saline (PBS) solution was added to the suspended culture and incubated at 4 °C for 15 min. Cultures were pelleted and rinsed twice with FACs buffer, followed by a 4 °C 15-min incubation with 0.2% saponin solution to permeabilize the cells. Cultures were centrifuged, rinsed twice and re-suspended in FC buffer following the permeabilization step. Fc receptor blocker (Miltenyi Biotec, 130-059-901), was added at a 1:5 dilution factor and incubated at 4 °C for 20 min. Antibodies were added at 0.1 to 10 μg/ml, incubated for 60 min at 4 °C, rinsed 2 × and re-suspended in FC buffer. The resultant cell solution was added to a 96-well plate and analyzed using a CytoFlex LX (Beckman Coulter) flow cytometer. The data was analyzed using CytExpert software, version 2.1 (Beckman Coulter).

### Immunocytochemistry and confocal microscopy

Mutant and WT cultures on glass coverslips were fixed with 4% PFA solution for 15 min and rinsed 3× with 1× PBS solution. Cells were then blocked with donkey serum blocking buffer (2.5 mL Donkey Serum + 2.5 mL BSA + 40 mL sterile water + 5mLs PBS 10x) for 1 h after a 15-min permeabilization period with 0.1% triton in PBS. Primary antibodies for MyoD, Pax7, desmin (Thermofisher Scientific PA5-16705, MyHC DSHB, A4.1025-s), and Ryanodine receptor (RyR) (Millipore, AB9078), dihydropyridine receptor (DHPR) (Thermofisher Scientific,MA3920) were added to blocked cultures and incubated overnight at 4 °C. The secondary antibodies donkey anti-mouse and anti-rabbit (Thermofisher Scientific, A10037, A-21206) were added the next day at a 1:250 dilution. Coverslips were rinsed 3× in 1× PBS mounted on glass slides with Prolong gold mounting medium with DAPI (Life Technologies P36931) after a 2-h incubation period for imaging. Images were taken with Axioskop 2 mot plus upright spinning disk confocal microscope (Carl Zeiss) that had been connected to a XCite 120 Fluorescence Illumination system (EXFO) beam with multi-spectral laser scanning software, Volocity version 6.3.0 (Perkin Elmer).

### Myotube phenotypic characterization

Five representative phase-contrast images per coverslip were taken from all myotube cultures with a Zeiss Axioscope 200. Images were loaded onto ImageJ software version 1.52 (NIH) and analyzed for average myotube number and myotube width using the cell counter plugin and straight free hand tool. Myotube width measurements were taken from phase contrast images as described by^52^. Briefly horizontal lines were drawn across the middle of the images, depending on direction of fused myotubes. Only widths of myotubes that crossed the line were measured using the ImageJ software. This was in effort to eliminate subjective data selection. The average length of myotubes from each line was estimated using the NeuronJ plugin of Image J. This ImageJ plugin has mostly been used for measuring axon lengths. Five randomized images per coverslip were taken of myotubes stained for MyHC. Myotubes in each field view were traced from one end to the other and measured. Myotube thickness was assessed by measuring the height of Z-stacks of MyHC-stained myotubes, using Volocity software, version 6.3.0 (Perkin Elmer). Fusion index was quantified for myotubes stained with MyHC and nuclear stain, 4′,6-diamidino-2-phenylindole, (DAPI). The number of nuclei within myotubes was divided by the amount of total nuclei to obtain a number between 0 and 1, where 0 means no fusion and 1 is fusion of all myoblasts^53–56^. Simply put,

$$Fusion \, index \, = \left( {\frac{number \,of \,DAPI - positive \,nuclei \,in \,myotubes \,in \,each \,field}{{total \,number \,of \,DAPI - positive \,nuclei \,in \,each \,field}}} \right).$$

### Myotube contraction assessment

After 14 days in differentiation medium, mutant and WT myotubes cultures in petri dishes were placed on a 37 °C heated stage (ThorLabs TC200) on an upright phase-contrast microscope (Zeiss Hal 100). Myotube contraction was elicited using silver electrode-driven, 1 V broad field stimulation at frequencies of 0.3, 0.5, 1.0, 2.0 and 4.0 Hz for 15 s. Myotube contractions were captured using a high-speed acquisition digital camera (Hamamatsu model C8484-05G) from a selected region of interest (ROI). This procedure is described in detail by Santhanam et al.^57^. Contraction synchrony was calculated by dividing the number of contractions by the total number of expected contractions at a given frequency.

### Myotube force and fatigability assessment

Mutant and WT iPSC-derived myoblasts were seeded at 400 cells/mm^2^ on collagen I-coated BioMEMs cantilever devices and assembled into engineered acrylic housings. The cultures were maintained in proliferation medium for 5 days, switched to DK-HI medium for 2 days, and then into terminal differentiation medium, N2, for 14 days. At the end of the differentiation period, the skeletal muscle myotube cultures were tested for contraction. As previously described, the cultures were transferred to a heated stage, equipped with a light source (above), and photodetector and laser (below). System control and data collection were performed as described by Wilson et al.^58^. Skeletal muscle from both mutant and WT cultures tested for their maximum contractile force output (MCF) with a frequency of 0.5 Hz and pulse width of 250 ms, at 5 V.

### Mitochondrial membrane potential assessment

Mutant and WT iPSC-derived myoblasts were plated at 50 cells/mm^2^ on Collagen I-coated 18 mm glass coverslips and differentiated into myotubes. At day 12 of differentiation, myotubes were incubated in 500 nM tetramethylrhodamine (TMRE) (Thermofisher Scientific, Cat# T669) solution for 30 min and rinsed twice with 1× PBS. Fluorescence images of the myotube cultures were taken with a Zeiss spinning disk confocal microscope (Axioskop 2 Mot Plus). Average fluorescence intensity per image was measured via ImageJ software.

### Mitochondrial metabolic profiling

Mitochondrial metabolism of mutant and WT iPSC-derived myotubes was assessed by measuring the rate of electron flux through the electron transport chain (ETC) following treatment with specific substrates. Assay solutions and preparation were done per manufacturer’s instructions (BiOLOG, Cat#s 14105, 72303, 74353). Briefly, muscle cultures were harvested using TrypLE Express and centrifuged at 280*g* for 5 min. The resulting pellet was re-suspended in 1× BiOLOG mitochondrial assay solution (MAS). The cell suspension was added to a 96-well plate with the various substrates, incubated in BiOLOG assay mix containing saponin, sterile water, MAS and BiOLOG’s redox dye MC. Data was collected over a two hour period, with optical density measurements taken every 5 min by the HT Synergy plate reader at 590 nm. Initial rate of each reaction of substrate was calculated from the resultant curves at the end of the experiment.

### Statistical analysis

Morphometry results were analyzed by One-Way ANOVA followed by Dunnett’s test. Functional data from the pixel subtraction apparatus was evaluated with ANOVA on ranks followed by the Holm-Sidak method. Cantilever experimental results were analyzed with One-Way ANOVA followed by Dunnett’s test.

## Supplementary information

Supplementary information

## Data Availability

The raw/processed data required to reproduce these findings cannot be shared at this time due to technical or time limitations.
