# Supplementary material for: Functional skeletal muscle model derived from SOD1-mutant ALS patient iPSCs recapitulates hallmarks of disease progression
Source: Sci Rep. 2020 Aug 31;10:14302. doi: 10.1038/s41598-020-70510-3 (PMC7459299; doi:10.1038/s41598-020-70510-3)
Supplement: Supplementary file 1 — Supplementary information [file 41598_2020_70510_MOESM1_ESM.docx]

**Supplementary Information**

**Title:** Functional Skeletal Muscle Model Derived from SOD1-mutant ALS Patient iPSCs Recapitulates Hallmarks of Disease Progression.

**Authors:** Agnes Badu-Mensah^1,2^, Xiufang Guo^1^, Christopher McAleer^3^, John Rumsey^3^, James J Hickman^1,3,*^

**Supplementary Figures**


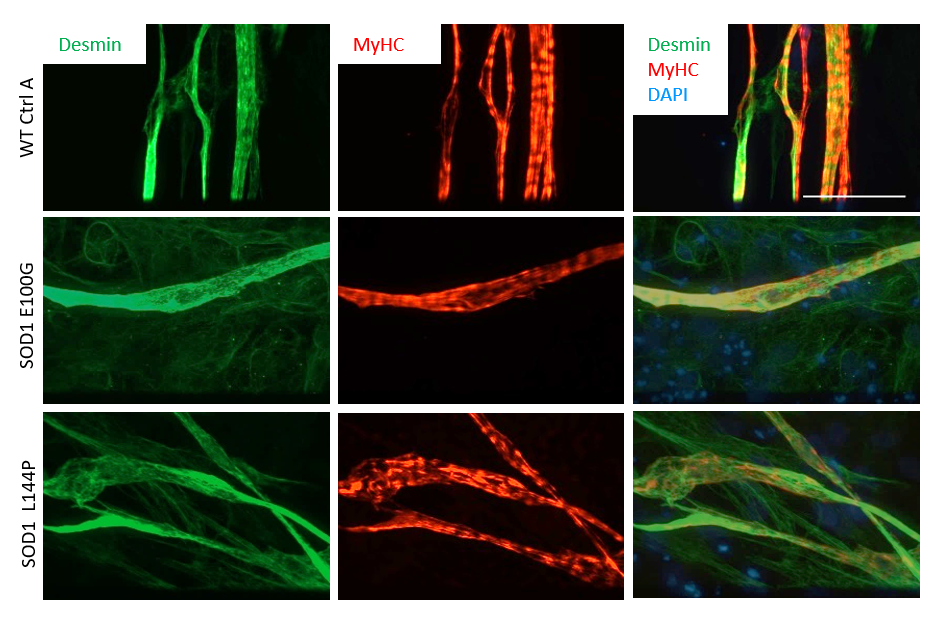


**Supplemental Figure S1. Phenotypic Myotube Characterization.** ICC images of myotube cultures stained for MyHC and Desmin. Notice that the unfused background cells in SOD1 cultures are Desmin^+^/MyHC^-^.


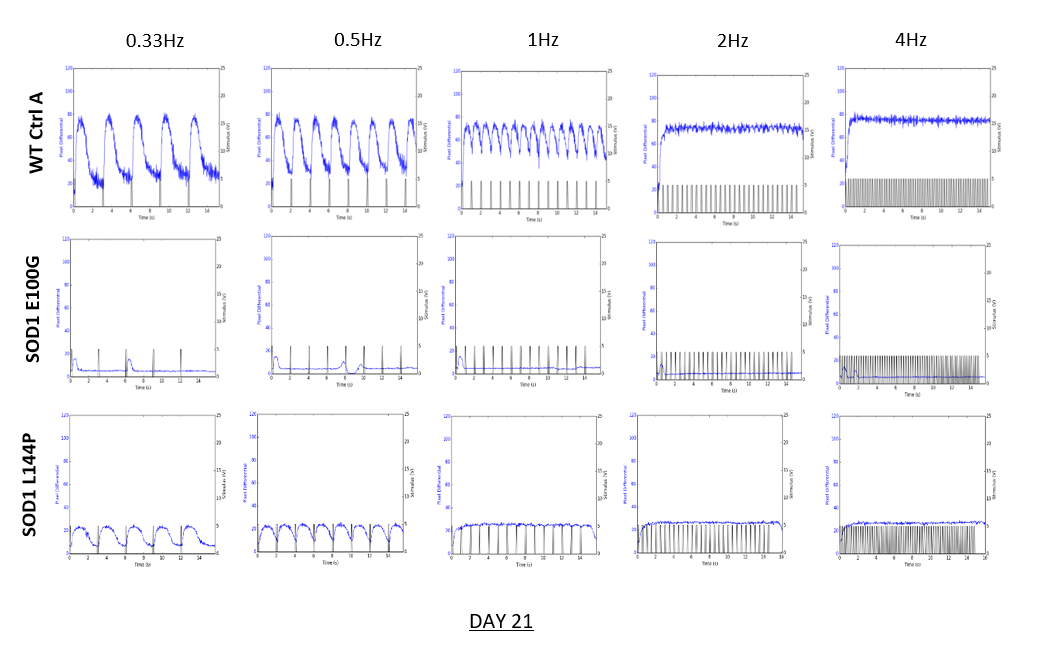


**Supplemental Figure S2. Myotube Functional Characterization.** Contraction traces of individual myotubes from iPSC-derived SOD1 E100G, SOD1 L144P and WT control cultures at 0.3, 0.5, 1, 2 and 4Hz on day 21.


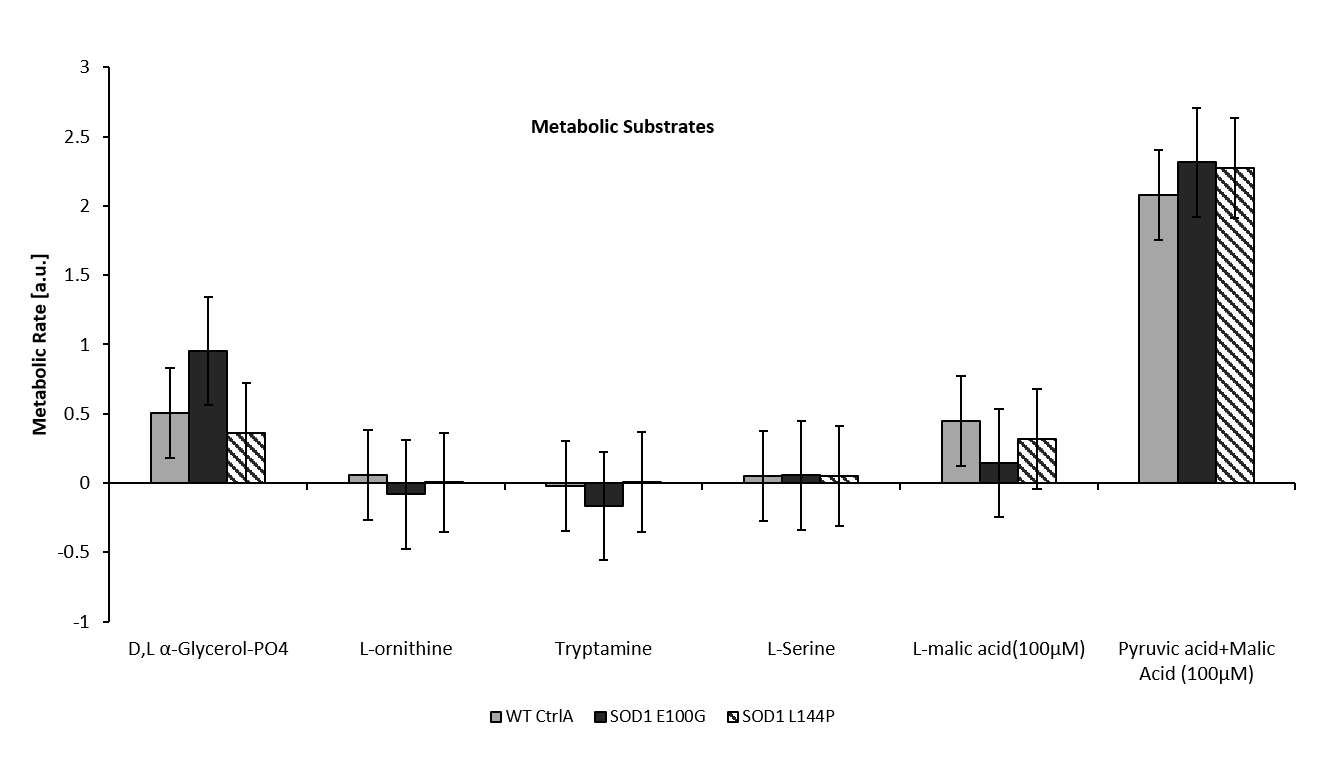


**Supplemental Figure S3. Phenotypic Myotube Characterization.** Other metabolic substrates tested during mitochondrial function assay.

| Preliminary Culture Optimization Tests | | | Results |
| --- | --- | --- | --- |
| Substrate | Matrigel | At least N(3) | A, F |
|  | Collagen I | At least N(3) | A, E |
| Proliferation/Expansion medium | Lonza | At least N(3) | A, B, F |
|  | Promocell-based | N(2) | C, F |
|  | DMEM/F12-based | N(2) | C, F |
|  | Myocult | N(2) | A, D, E |
| Differentiation medium | DK-HI – N2 | N(2) | A, B, F |
|  | N2 | N(2) | A, B, F |
|  | DK-HI +Dex – N2+Dex | N(2) | A, D, E |
|  | DK-HI +Dex – N2+IGF | N(1) | A, B, F |
|  | DK-HI + Dex – N2+Dex+ IGF | N(1) | A, B, F |

**Supplemental Table S1. Preliminary culture optimization Experiments.** Table of substrates, medium formulations and factors that were tested during the protocol optimization phase of the project. IGF: Insulin-like Growth Factor; Dex: Dexamethasone; Results Legend: A. Supports myoblast fusion; B. Lower myoblast fusion; C. Did not support myoblast fusion; D. Yielded enhanced myoblast fusion; E. Pursued and F. Did not pursue.
